# Sexual and Reproductive Health Literacy and Service Utilization Among Young People in Southwest Nigeria

**DOI:** 10.1177/23779608251411367

**Published:** 2026-01-27

**Authors:** Aanuoluwapo Omobolanle Olajubu, Abiola Olubusola Komolafe, Adesegun Olayiwola Fatusi

**Affiliations:** 1 Department of Nursing Science, 486455 Obafemi Awolowo University, Ile-Ife, Nigeria; 2 Department of Community Medicine, Universit of Medical Sciences, Ondo, Nigeria

**Keywords:** sexual health, reproductive health, service utilization, sexual and reproductive health literacy, young people, Nigeria

## Abstract

**Background:**

Young people encounter numerous health challenges, including sexual and reproductive health (SRH) problems. Poor SRH literacy plays a key role in young people's risky sexual behavior, which can lead to untoward SRH-related health outcomes.

**Objectives:**

This study assessed the level of SRH literacy and SRH service utilization among in-school and out-of-school young people in Osun state, southwest Nigeria

**Methods:**

A descriptive cross-sectional study among 1,096 young people aged 15–24 in two universities and one community in Osun state. Respondents were selected using a multi-stage sampling process. A structured questionnaire incorporating the health literacy questionnaire (HLQ) was used to collect data, which was analyzed using descriptive and inferential statistics.

**Results:**

The mean age was 19.0 ± 2.6 years; about half (56.5%) were females, while about one-third (35.2%) were sexually experienced. The mean literacy score was 56.6 ± 17.2, and 79.5% had low SRH literacy levels. Only 12.9% utilized any SRH-related care or service. SRH information and counselling were the most utilized services (78.7%), while the least was cervical cancer screening (11,4%). A lower SRH literacy score was significantly associated with being out of school (*P* < .001) and non-use of SRH services (*P* < .001). Respondents with good literacy levels were three times more likely to have accessed and utilized SRH care and services (OR: 3.08, 95% CI: 1.99–4.76).

**Conclusion:**

The respondents had a low SRH literacy level, which was associated with poor utilization of SRH services. Appropriate interventions are required to improve SRH literacy levels in the study setting.

## Introduction

Young people aged 10–24 account for about 25% of the world's population, and they constitute 32% of the population in low- and middle-income countries (United Nations Foundation, 2024). People in this age group are prone to certain sexual and reproductive health (SRH) problems, such as unsafe abortion, young maternal death, violence, and sexually transmitted infections (STIs), including HIV/AIDS (Dabiri et al., 2019; Envuladu et al., 2022; Vongxay et al., 2019). Adverse sexual and reproductive health outcomes among young people continue to pose significant public health challenges in numerous countries, including Nigeria (Agu et al., 2024; Amanu et al., 2023; Envuladu et al., 2022; Hassan & Golub, 2025; Kelecha et al., 2024; Mukeshimana et al., 2025; Nkrumah et al., 2025; Vongxay et al., 2019).

An estimated 1.7 million adolescents were living with HIV in 2021, with around 90% in the WHO African Region (World Health Organization, 2024). In developing nations, at least 777,000 girls under the age of 15 and about 12 million girls between the ages of 15 and 19 give birth annually. One of the main causes of death for females between the ages of 15 and 19 worldwide is complications related to pregnancy and childbirth (Pons-Duran et al., 2021; World Health Organization, 2024). Inadequate SRH literacy and low utilization of the available SRH facilities for quality services have been identified as contributors to most SRH problems and mortality among young people (Agu et al., 2021; Envuladu et al., 2022).

## Review of Literature

Health literacy is a multidimensional concept. It is not limited to the level of knowledge or the amount of information an individual has regarding their health or a particular health subject. Rather, it encompasses the ability of the individual to access and obtain relevant and qualitative information regarding their health, properly process and understand the information, and utilize the knowledge to make informed decisions that promote optimal health (Liu et al., 2020; Vongxay et al., 2019). Sexual and reproductive health literacy thus refers to the capacity of an individual to access, acquire, and comprehend pertinent SRH information and knowledge and apply the same to make decisions that will best promote sexual and reproductive well-being (Vongxay et al., 2019).

Studies have reported poor SRH literacy among young people in low- and middle-income countries such as Nigeria (Agu et al., 2024; Amanu et al., 2023; Debella et al., 2024; Leekuan et al., 2022; Mugabi et al., 2023; Mukeshimana et al., 2025; Nkrumah et al., 2025; Okooboh & Martins, 2023). This inevitably leads to poor SRH decisions, such as unprotected sexual intercourse and unsafe abortion, which may subsequently result in adverse social and health outcomes (Agu et al., 2021; Melesse et al., 2020; Ross et al., 2021; Vongxay et al., 2019). The importance of adequate SRH literacy cannot be overemphasized; these include making the right SRH-related decisions, good health-seeking behavior, increased utilization of SRH services, and improved health outcomes, among others (Agu et al., 2021; Leekuan et al., 2022).

The utilization of SRH services and facilities among young people in sub-Saharan Africa is critical to public health. Despite the availability of these services, there are significant barriers to access, including stigma, lack of awareness, and cultural norms (Agu et al., 2021; Envuladu et al., 2022; Nartey et al., 2025; Ninsiima et al., 2021; Olajubu et al., 2025). Consequently, many young individuals face challenges in accessing essential SRH services such as contraception, STI testing, and reproductive health education. Addressing these barriers requires comprehensive strategies that prioritize youth-friendly services, community engagement, and education to empower young people to make informed choices about their sexual and reproductive health (Nartey et al., 2025; Olajubu et al., 2025; Wakjira & Habedi, 2022).

A systematic review found that the level of young people's SRH literacy in sub-Saharan Africa is a cause for concern and has not received enough attention in the literature (Amanu et al., 2023). The authors emphasized that designing and implementing effective interventions to enhance SRH literacy and health outcomes among this demographic requires a deeper understanding of the issue, taking into account diverse social and cultural contexts. Although previous studies in Nigeria have investigated SRH knowledge and service utilization among young people, most have concentrated narrowly on knowledge-based measures (Adione et al., 2023; Ijadunola et al., 2023; Ninsiima et al., 2021; Ugwu et al., 2022; Utaka et al., 2023). These studies have largely overlooked the broader, multidimensional construct of SRH literacy. Moreover, none, to the best of available evidence, have compared SRH literacy levels between in-school and out-of-school young persons, who may differ significantly in their exposure to information and access to services. Consequently, empirical evidence remains limited on how these variables interact within the sociocultural context of the region. This study, therefore, assessed the level of SRH literacy and SRH service utilization among in-school and out-of-school young people in Osun state, southwest Nigeria.

## Methods

### *Study Design and Setting*

This study employed a descriptive cross-sectional research design to assess SRH literacy and service utilization among young people in Osun state, southwestern Nigeria. It was conducted between January and March 2023 to provide the baseline data for the Sexual Health Literacy (SHELTER) project, designed to elucidate and address gaps in SRH literacy among in- and out-of-school young adults in Osun state. There are six states in the southwestern region, each of which is subdivided into Local Government Areas (LGAs), with three-tiered (primary, secondary, and tertiary) healthcare facilities, offering full SRH services. The public and private universities also have health centers that provide basic healthcare services, including SRH counselling and care, with well-established referral systems to higher levels of care, where and when necessary.

### *Study Population and Sample*

The sample was selected from in-school young people (15–24 years) from private and public universities and out-of-school young people from the community. Multiple sampling techniques were used in the selection of the participants. Osun state was randomly selected out of six states in southwestern Nigeria.

#### *In-School Participants.*

The universities in the state were stratified into public and private institutions, out of which two - one public, i.e., Osun State University, Osogbo campus (UNIOSUN), and one private, i.e., Redeemer's University, Ede (RUN) -were randomly selected. The two universities were stratified into faculties, out of which two faculties were randomly selected from each of the two campuses. All the departments in the selected faculties were proportionately sampled. In each department, respondents were recruited using convenience sampling.

#### *Out-of-School Participants.*

Four communities in Ilesha West Local Government Area were selected through a multi-stage sampling technique. In the households and streets of the communities, consenting young people, aged 15–24, were conveniently recruited until the sample size was filled.

#### *Inclusion and Exclusion Criteria.*

Individuals within the age range of 15–24 years, who were full-time students of the selected universities, who provided informed consent to participate in the study, were included as in-school participants. For out-of-school participants, individuals who resided in the selected community, who were not enrolled in any formal educational institution, and had not completed a tertiary education at the time of the study, were included. Those who had any form of disability that may interfere with comprehension of the questionnaire were excluded.

#### *Sample Size.*

For each of the three study sites, a sample size of 400 was estimated based on a previously reported 65% non-utilization rate of SRH services. (Onyeneke et al., 2021) and a 10% non-response rate, yielding a total sample of 1,200. In each of the three settings, 400 participants were recruited. However, a total of 1,096 questionnaires (761 in-school and 335 out-of-school) were retrieved with a response rate of 91.3%.

### *Ethical Considerations*

Ethical approval for the study was obtained from the Health Research Ethics Committee (HREC) of the author's institution. Permission to conduct the study was secured from relevant authorities, and written informed consent was sought from each participant. Young adults between the ages of 15 and 17 years were regarded as emancipated minors in this study, as participation posed no associated health risk (Munung et al., 2022).

### *Data Collection*

Data were collected with the aid of a structured self-administered questionnaire, which had three sections: (a) socio-demographic information, (b) Health Literacy Questionnaire (HLQ), and (c) SRH service utilization.

#### *Health Literacy Questionnaire.*

SRH literacy was assessed with an adapted version of the Health Literacy Questionnaire (HLQ), a standardized and validated multidimensional tool. It contains a total of 44 items and measures nine independent domains, which describe the ability of individuals to access, understand, and make effective use of health information and services (Batterham et al., 2016; Budhathoki et al., 2022; Osborne et al., 2013).

The HLQ has been used in many countries and different contexts, including population surveys and has been subjected to validity testing across various demographics. (Anwar et al., 2020; Ayamolowo et al., 2020; Batterham et al., 2016; Beauchamp et al., 2015; Budhathoki et al., 2022; Mbada et al., 2022; Said Bodur et al., 2017). It has undergone psychometric evaluation and has been used in previous studies in southwest Nigeria, yielding good composite reliability and Cronbach's alpha of ≥0.7 across all the scales (Ayamolowo et al., 2020; Mbada et al., 2022). For this study, the items were adapted specifically to sexual and reproductive health. For example, Item 1 which read “I feel I have good information about health” was rephrased as “I feel I have good information about sexual and reproductive health” Another item “I know how to find out if the health information I receive is right or not” was rephrased to read “I know how to find out if the sexual and reproductive health information I receive is right or not.” The questionnaire was pilot-tested among 50 young people in Ife Central LGA. The questions were easily understood, and the reliability test yielded a Cronbach alpha of 0.92 for the HLQ scale.

The HLQ has nine scales. The first five scales had 23 items, which were scored on a 4-point Likert-type response (1 = *strongly disagree*, 2 = *disagree*, 3 = *agree*, and 4 = *strongly agree*). The five scales are: “feeling understood and supported by SRH care providers” (four items); “having sufficient information to manage SRH” (four items); “actively managing my SRH health” (five items); “social support for SRH” (five items); “appraisal of SRH information” (five items). The remaining four scales have 21 items and they assess the ease or difficulty with which individuals can access, understand, and use SRH information and services. These include “ability to actively engage with healthcare providers” (five items); “navigating the healthcare system” (six items); “ability to find good SRH information” (five items); “understanding SRH information well enough to know what to do” (five items).

For each of the nine scales, a mean score is computed for each respondent by adding the item scores and dividing by the number of items in the scale. A composite SRH health literacy score was also computed by adding all the scores in the 44 items. The total scores were thereafter converted to percentage scores, which were dichotomized into poor (scores < 70%) and good SRH literacy (Ayamolowo et al., 2020).

The service utilization section asked if respondents had ever visited a healthcare provider or facility to access any SRH service, such as contraceptive counselling and/or uptake, voluntary counselling and testing (VCT) for HIV, cervical cancer screening, vaccination, screening or treatment for STIs, abortion or post-abortion care, pre-marital counselling or screening, pre-natal or ante-natal care or any other SRH-related counselling or care.

### *Study Variables*

#### *SRH Literacy.*

This is defined as an individual's capacity to obtain, understand, and use SRH-related information to make informed decisions regarding their reproductive health. It was assessed using the HLQ as described above. Each respondent had scale scores computed as earlier described, and a total HLQ score in percentage. To dichotomize into good and poor SRH literacy, those with scores ≥ 70% were categorized as having good health literacy (Ayamolowo et al., 2020).

#### *SRH Service Utilization.*

An individual was categorized as having utilized SRH service if they had sought and received any of the SRH services listed above from a healthcare provider.

### *Biases*

Efforts were made to minimize bias at every stage of the study. Institutions and faculties were randomly selected, and all departments within the selected faculties were proportionately sampled to ensure representativeness. Nonetheless, the possibility of recall and social desirability biases cannot be completely ruled out. To mitigate these, respondents were assured of anonymity and strict confidentiality, and they were encouraged to provide honest responses. In addition, the questionnaire was self-administered in a non-judgmental setting to reduce pressure toward socially acceptable answers and to enhance the accuracy of self-reported information.

### *Data Analysis*

Data were entered into and analyzed with SPSS 26. Categorical variables were summarized with frequencies and percentages, while means with standard deviations were presented for variables. The difference in mean HLQ scores between those who had utilized SRH services and those who had not was assessed by the independent *t*-test. Cohen's *d*-statistics were computed as the measure of the effect sizes (ES) for standardized differences in means between the groups. The association between categorical variables was determined with the use of the Pearson’s chi-square. Variables that were significantly associated with the utilization of SRH services were entered into a multivariable logistic regression model.

## Results

### *Sociodemographic and Baseline Characteristics*

There were 1,096 respondents (91.3% response rate) among whom 761 (69.4%) were in-school, and 335(30.6%) were out-of-school. The mean age was 19.0 ± 2.6 years, with a higher proportion (58.7%) being in the age group 15–19 years. More than half (619, 56.5%) were females, while more than 99% had completed at least secondary school. About one-third (386, 35.2%) were sexually experienced.

### *SRH Literacy*

The mean scores in each of the HLQ scales are highlighted in Table 1. The highest mean score was recorded in the last domain (*understand SRH information well enough to know what to do*) (3.59 ± 0.95), while the lowest score (2.50 ± 0.66) was reported in the first domain (*feeling understood and supported by SRH care providers*) scale. The composite scores were computed, and after conversion to percentages, the mean overall HLQ score was 56.6 ± 17.2%. Among the participants, 871 (79.50%) had poor SRH literacy, while 20.50% had good SRH literacy.

**Table 1.** Health Literacy Questionnaire (HLQ) Mean Scale Scores for Respondents (*N* = 1,096).

HLQ scales (adapted for SRH)		*M* (*SD*)
		Range: 1 (*lowest*) to 4 (*highest*)
1	Feeling understood and supported by SRH care providers	2.50 (0.66)
2	Having sufficient information to manage my SRH	2.68 (0.63)
3	Actively managing my SRH	2.80 (0.58)
4	Social support for SRH	2.82 (0.59)
5	Appraisal of SRH information	2.60 (0.60)
		Range: 1 (*lowest*) to 5 (*highest*)
6	Ability to actively engage with SRH care providers	3.08 (1.02)
7	Navigating the healthcare system	3.18 (0.98)
8	Ability to find good SRH information	3.30 (0.99)
9	Understand SRH information well enough to know what to do	3.59 (0.95)

Table 2 shows the association between SRH literacy and the categories of various demographic and sexual characteristics of respondents. There was a significant association between SRH literacy and each of the following: schooling status (*P* = .04), number of sexual partners (*P* = .01), and age at coitarche (*P* = .01).

**Table 2.** Relationship Between Sexual and Reproductive Health Literacy and Respondents’ Characteristics.

Variable	Frequency (%)	Poor literacy level *n* (%)	Good literacy level *n* (%)	Test statistics χ^2^/*r*	*P*-value
Age category					
15–19	643 (58.7)	518 (80.6)	125 (19.4)	1.31	.29
20–24	453 (41.3)	353 (77.9)	100 (22.1)		
Sex					
Female	619 (56.5)	496 (80.1)	123 (19.9)	0.38	.54
Male	477 (43.5)	375 (78.6)	102 (21.4)		
Highest education					
Primary/secondary	1049 (95.7)	834 (79.5)	215 (20.5)	0.17	.89
Postsecondary	47 (4.3)	37 (78.7)	10 (21.3)		
Religion					
Christian	931 (84.9)	742 (79.7)	189 (20.3)	0.20	.66
Islam-others	165 (15.1)	129 (78.2)	36 (21.8)		
Family type					
Monogamous	927 (84.6)	729 (78.6)	198 (21.4)	2.54	.11
Polygamous	169 (15.4)	142 (84.0)	27 (16.0)		
Schooling status					
In-school	761 (69.4)	587 (77.1)	174 (22.9)	8.32	.004
Out-of-school	335 (30.6)	284 (84.8)	51 (15.2)		
Smartphone ownership					
No	101 (9.2)	83 (82.2)	18 (17.8)	0.50	.48
Yes	995 (90.8)	788 (79.2)	207 (20.8)		
Ever had sex					
No	710 (64.8)	559 (78.7)	151 (21.7)	0.674	.41
Yes	386 (35.2)	312 (80.8)	74 (19.2)		
Sexual partners					
0	732 (66.8)			−0.07^a^	.01
1	236 (21.5)				
>1	128 (11.7)				
Age at Coitarche					
<10	8 (0.7)			0.08^a^	.01
10–14	41 (3.7)				
15–19	239 (21.8)				
20–24	97 (8.9)				

a Spearman rho.

### *Utilization of SRH Services*

**Figure 1.**
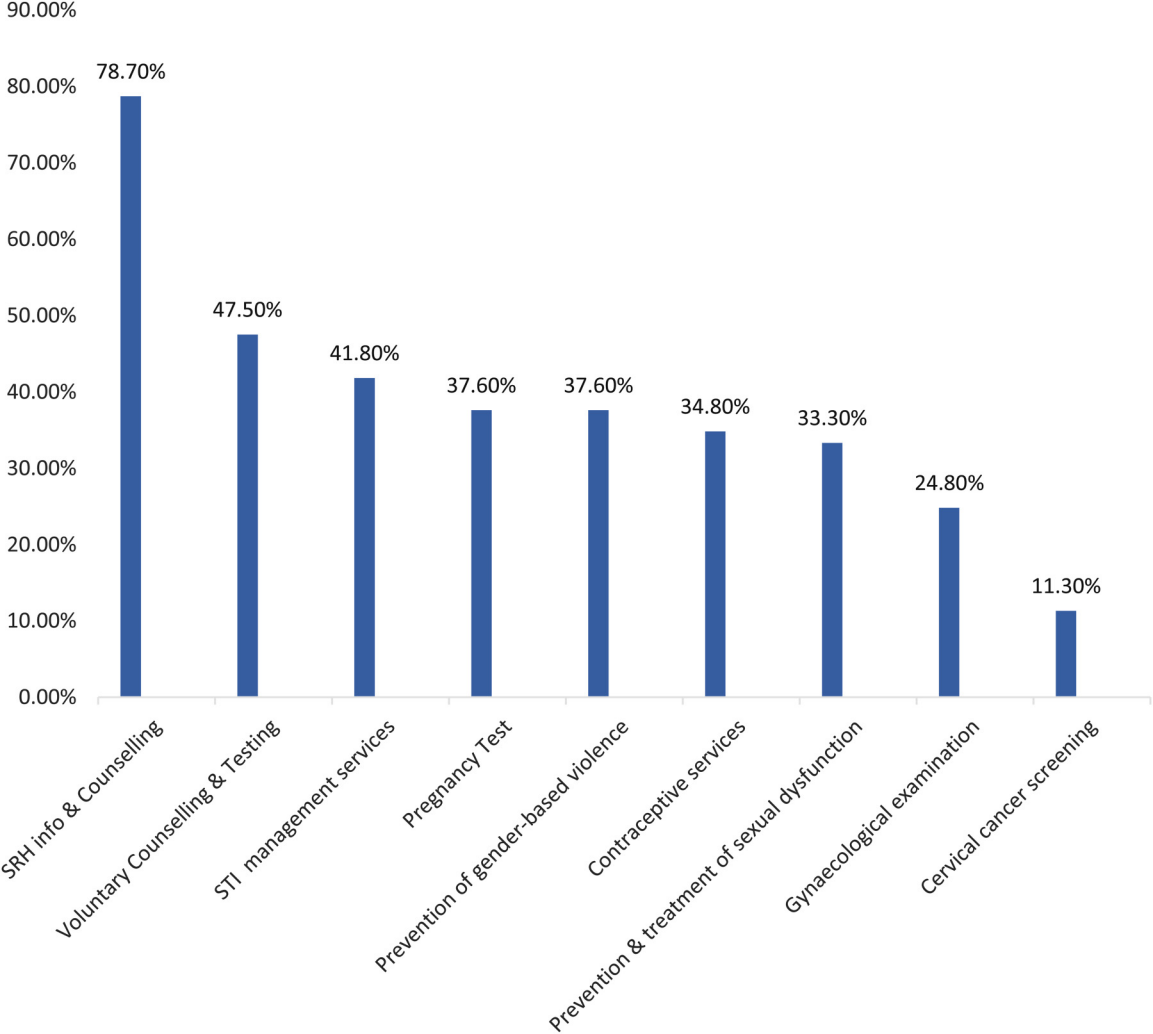
Utilization of SRH services.

Few participants (141; 12.9%) had accessed and utilized some form of SRH care or service at one time or another. Among those who utilized services, 111(78.7%) utilized SRH information and counselling, followed by voluntary counselling and testing (*n* = 67; 47.5%), sexually transmitted infection screening, diagnosis, and management services (*n* = 59; 41.8%), while the least utilized service was cervical cancer screening (*n* = 16; 11.4%). (Figure 1)

Table 3 shows the difference in the mean HLQ scale scores between those who had a history of utilizing SRH services and those who did not. Those who utilized SRH service had significantly higher SRH literacy scores in all the domains of the HLQ (*P* < .05) except in domain four, i.e., “social support for SRH” (*P* = .088). The effect size of the differences was highest in the first scale, i.e., “feeling understood and supported by healthcare providers,” while it was lowest for the fourth scale (in which the difference was not statistically significant).

**Table 3.** Relationship Between HLQ Scale Scores and SRH Service Utilization.

HLQ scale	Service utilization		*t*-value	*P*-value	Effect size (Cohen's *d*)
	Yes *M* (*SD*)	No *M* (*SD*)			
HLQ Scale 1	2.75 (0.65)	2.46 (0.66)	4.87	.000	0.44
HLQ Scale 2	2.81 (0.60)	2.66 (0.63)	2.70	.007	0.24
HLQ Scale 3	2.92 (0.62)	2.78 (0.57)	2.71	.007	0.24
HLQ Scale 4	2.90 (0.65)	2.81 (0.58)	1.71	.088	0.15
HLQ Scale 5	2.81 (0.58)	2.57 (0.60)	4.52	.000	0.40
HLQ Scale 6	3.43 (0.92)	3.03 (1.03)	4.37	.000	0.39
HLQ Scale 7	3.46 (0.90)	3.14 (1.00)	3.54	.000	0.32
HLQ Scale 8	3.53 (0.93)	3.26 (1.00)	3.00	.003	0.27
HLQ Scale 9	3.76 (0.85)	3.57 (0.96)	2.30	.021	0.21

Table 4 highlights the association between SRH services utilization and the overall level of SRH literacy, along with other categorical variables. There was a significant association between good SRH literacy and higher prevalence of utilization (*P* < .001). Other factors that were significantly associated with a higher utilization of SRH services include older age (*P* < .001), female gender (*P* = .039), postsecondary education (*P* = .002), those who have ever had sex (*P* < .001), being in a cohabiting relationship (*P* < .001) and higher number of sexual partners (*P* < .001).

**Table 4.** Association Between Utilization of Sexual and Reproductive Health Services and Respondents’ Characteristics.

	Utilization No, *n* (%)	Utilization Yes, *n* (%)	χ^2^	*P*-value
SRH literacy				
Poor	779 (89.4)	92 (10.6)	20.06	<.001
Good	176 (78.2)	49 (21.8)		
Age category				
15–19	593 (92.2)	50 (7.8)	35.94	<.001
20–24	362 (79.9)	91 (20.1)		
Gender				
Female	528 (85.3)	91 (14.7)	4.28	.039
Male	427 (89.5)	50 (10.5)		
Highest level of education				
Primary	5 (83.3)	1 (16.7)	12.66	.002
Secondary	917 (87.9)	126 (12.1)		
Postsecondary	33 (70.2)	14 (29.8)		
Tribe				
Yoruba	820 (86.6)	127 (13.4)	1.85	.396
Igbo	68 (90.7)	7 (9.3)		
Hausa	67 (90.5)	7 (9.5)		
Family type				
Monogamous	815 (87.9)	112 (12.1)	3.29	.070
Polygamous	140 (82.8)	29 (17.2)		
Sexual partners				
0	691 (94.4)	41 (5.6)	118.80	<.001
1	183 (77.5)	53 (22.5)		
>1	81 (63.3)	47 (36.7)		
Schooling status				
In-school	673 (88.4)	88 (11.6)	3.76	.052
Out-of-school	282 (84.2)	53 (15.8)		
Relationship status				
Not in a relationship	647 (90.6)	67 (9.4)	31.96	<.001
Cohabiting	11 (57.9)	8 (42.1)		
In a relationship but not living together	299 (81.8)	66 (18.3)		
Ever had sex				
No	673 (94.8)	37 (5.2)	105.35	<.001
Yes	282 (73.1)	104 (26.9)		
Smartphone ownership				
No	85 (84.2)	16 (15.8)	0.88	.348
Yes	870 (87.4)	125 (12.6)		

As shown in Table 5, after controlling for the factors that were significantly associated with utilization, SRH literacy maintained its significance as a predictor of the utilization of SRH services. Those who had a good literacy level were three times more likely to have accessed and utilized some SRH service (OR: 3.08, 95% CI: 1.99–4.76). Other predictors in the study include female gender (OR: 2.10. 95% CI: 1.35–3.28), history of sexual activity (OR: 3.91, 95% CI: 1.28–11.96), and having two or more sexual partners (OR: 3.75, 1.20–11.70).

**Table 5.** Predictors of SRH Service Utilization Using Multivariate Logistic Regression.

Variable	Adjusted odds ratio	95% CI	*P*-value
SRH literacy			
Poor	1		
Good	3.08	1.99–4.76	.000
Age category			
15–19	1		
20–24	1.28	0.82–1.99	.277
Gender			
Male	1		
Female	2.10	1.35–3.28	.001
Highest level of education			
Primary	1		
Secondary	0.36	0.03–3.91	.401
Postsecondary	0.74	0.62–8.77	.810
Sexual partners			
0	1		
1	1.22	0.40–3.76	.724
>1	3.75	1.20–11.70	.023
Ever had sex			
No	1		
Yes	3.91	1.28–11.96	.017
Relationship status			
None	1		
Cohabiting	2.32	0.74–7.27	.148
In a relationship but not living together	0.81	0.51–1.29	.378
Schooling status			
In school	1		
Out-of-school	1.05	0.68–1.63	.830

## Discussion of Findings

Sexual and reproductive health is an essential aspect of overall well-being. Moreover, SRH literacy plays a vital role in preventing SRH diseases and promoting healthy, fulfilling relationships and sexual experiences. This study assessed the SRH literacy level and the utilization of SRH services among young people.

The majority (79.5%) of participants in this study were categorized as having poor sexual and reproductive health literacy, hence indicating a considerable SRH literacy gap. This finding revealed that young people had poor SRH literacy, which is consistent with previous studies on SRH literacy (Dabiri et al., 2019; Lee et al., 2022; Mugabi et al., 2023; Vongxay et al., 2019), especially in developing countries, including Nigeria. Diverse factors such as poor or inadequate comprehensive sex education, cultural taboos, limited access to accurate information, social stigma, and lack of open discussion about SRH could be responsible for poor SRH literacy among the participants in this study. Also, the knowledge gap could suggest broader challenges within the education system, cultural norms, and social attitudes. All these factors need to be addressed through comprehensive sex education, open dialogues on sexuality and matters relating to reproductive health, and accessible resources to improve overall health literacy. Contrary to these findings were studies (Jamali et al., 2022; Panahi et al., 2021) conducted among women in Iran, where only a few participants had poor SRH literacy. This discrepancy could be a result of the fact that participants in the two studies were only women and their ages ranged from 15 to 45 years; these could have increased their SRH literacy level, since being a female and older age have been reported to be associated with higher SRH literacy (Simpson et al., 2015).

Statistical analysis revealed a significant association between SRH literacy and each of the following: schooling status, number of sexual partners, and age at coitarche. It is not unusual that in-school participants exhibited higher SRH literacy; this could be because in-school participants had ongoing access to formal education programs and SRH materials, as well as better opportunities to discuss SRH topics with peers. A previous study reported that out-of-school adolescents had limited access to SRH information and services due to sociocultural barriers, health system barriers, and inadequate exposure to relevant educational materials (Hailemariam et al., 2021).

The statistically significant relationship between the number of sexual partners and SRH literacy revealed that sexual activity is a strong factor influencing SRH literacy. Participants with no sexual partner had higher SRH literacy scores, followed by participants with a single sexual partner, while participants with more than one sexual partner had the lowest SRH literacy score. Several factors could be responsible for this finding, such as education and awareness. Participants with higher SRH literacy scores might prioritize education and awareness about sexual health and relationships, which could lead to a focus on fewer sexual partners as they seek informed and responsible choices. Also, cultural norms and values around sexual behavior and relationships vary; some cultures might encourage monogamy, while others might have different views on multiple partners. The knowledge about sexually transmitted infections and other health risks associated with multiple partners might influence individuals to choose fewer sexual partners. This suggests that the improvement of SRH literacy may potentially promote healthier sexual behavior among young persons.

In the same vein, the fact that participants who had their first sexual experience at a younger age had lower SRH literacy scores suggests that higher SRH literacy encourages individuals to delay the age of first sexual contact (Hubert et al., 2021; Vanwesenbeeck et al., 2016). In terms of utilization of SRH care or services, very few young people (12.9%) in this study had ever accessed and utilized some form of SRH care or services. This result is similar to the low rate of utilization reported in some previous studies among young persons (Abdurahman et al., 2022; Agu et al., 2021; Binu et al., 2018; Envuladu et al., 2022). Conversely, the study of Utaka et al., (2023) found that 63.7% of the respondents had ever utilized SRH services.

The poor utilization of SRH services by the participants in this study could be influenced by various factors, such as poor SRH literacy, lack of access to healthcare facilities, poor attitude of healthcare providers, stigma around discussing sexual health, and the level of support from family and peers. Therefore, insights from this study accentuate the importance of addressing the diverse factors influencing the utilization of SRH services among young people. This information can guide the development of strategies to enhance awareness, accessibility, and acceptance of these services among this group of people, ultimately promoting better sexual and reproductive health.

Among those who utilized services, the majority reported utilization of SRH information and counseling, while cervical cancer screening was the least utilized service. This finding is in congruence with the findings from the study of Abdurahman et al., (2022), where the majority of their respondents also utilized SRH information, education, and counselling. Less than half utilized voluntary counselling and testing (VCT), which is in alignment with findings from previous studies, where less than half of the respondents sought VCT (Utaka et al., 2023; Wakjira & Habedi, 2022). The variations in utilization of different SRH services could be influenced by factors such as awareness, educational levels about the services, cultural beliefs, accessibility, socio-economic status, stigma, and perceived risk (Envuladu et al., 2022; Ninsiima et al., 2021). As previously reported (Ayamolowo et al., 2020; Mafiana et al., 2022; Petersen et al., 2022), cultural sensitivity, discomfort, ignorance, and privacy concerns may have contributed to cervical cancer screening being the least used service by respondents in this study.

Inferential analysis revealed that a good level of SRH literacy was significantly associated with a higher prevalence of utilization of SRH services. Those with good SRH literacy had a higher utilization rate (21.8%) than those with poor SRH literacy (10.6%), which is consistent with the findings from another Nigerian study (Ijadunola et al., 2023). This is not unexpected, as greater knowledge or literacy on a specific health issue generally leads to better health-seeking behavior. (Abdurahman et al., 2022; Leekuan et al., 2022). In the context of this study, health literacy is the capacity to access, understand, appraise, and apply health information. Hence, individuals with higher SRH literacy are more likely to know how to access the necessary SRH care. However, there was a contrary finding in the study of Utaka et al., (2023) where there was no association between the utilization of SRH services and good SRH knowledge. The utilization of SRH care services in this study was also found to be significantly associated with other variables such as older age, being a female, postsecondary level of education, ever had sex, being in a cohabiting relationship, and having a higher number of sexual partners.

However, multivariate analysis showed that the independent predictors of SRH service utilization include having good health literacy, being female, a history of sexual activity, and having multiple sexual partners. Those with good literacy levels were three times more likely to have accessed and utilized one form of SRH service or the other. Another study reported that higher SRH literacy was associated with a higher likelihood of seeking SRH care services, which is consistent with the findings in this study (Väisänen et al., 2021).

Among the few who utilized SRH care and services, a greater proportion were females. This could be due to societal expectations and gender roles, which make men think that women should be the ones to focus on SRH issues (Adione et al., 2023; Roudsari et al., 2023). This might influence women to prioritize their reproductive health and seek medical advice more than men. This disparity may reflect multifaceted socio-cultural constructs, necessitating gender-sensitive strategies for enhancing male engagement in SRH services (Roudsari et al., 2023).

Respondents with a history of sexual activity exhibited markedly higher utilization rates in comparison to those without such experience. This was similar to previous findings where respondents who have ever had sex were more likely to utilize SRH services compared to those who had never had sex (Envuladu et al., 2022; Utaka et al., 2023). The reason for the positive association could be that the respondents who have ever had sex have more perceived needs for SRH services than those who have never had sex (Utaka et al., 2023). Also, sexual activity could lead to potential health issues, such as STIs or unintended pregnancies, which could prompt individuals to seek SRH services for prevention, testing, and treatment. The individuals with multiple sexual partners displayed higher service utilization, which may underscore the fact that having multiple sexual partners predisposes one to various SRH issues, such as STIs, including HIV/AIDS, among others, which could have made the participants seek SRH services compared to those who practice abstinence or safe sex. However, this is contrary to findings from the study of Ijadunola et al., (2023), where respondents with single or no sexual partners utilized SRH services more than respondents with multiple sexual partners. Many respondents in their study had good knowledge of SRH, which could have translated into the utilization of SRH services and behavior of abstinence and fidelity, as shown in their results.

### *Strengths and Limitations*

The findings in this study highlight the need for interventions to improve SRH literacy and service utilization among young people in the study area. The findings should be interpreted with consideration of certain potential limitations. These include the possibility of self-reporting bias, which may be especially relevant due to the sensitive nature of SRH information. Participants may not have disclosed accurate details about their behaviors or service utilization, influenced by embarrassment or fear of judgment. Additionally, the self-reported nature of the data could have introduced recall bias. However, efforts were made to mitigate these concerns. Participants were assured of the confidentiality of their information, which may have encouraged more honest and accurate responses. They were also assured that their responses would be free from judgment and that their privacy would be fully respected. These measures helped to create a more comfortable and trusting environment for participants, potentially minimizing the impact of self-reporting bias on the findings. Generalizability is another limitation, as findings from Osun state may not fully apply to other Nigerian regions due to socio-cultural and economic differences that could influence SRH behaviors. Furthermore, the use of a cross-sectional design limits the ability to establish causal relationships between SRH literacy and service utilization among young people.

The study, however, had important strengths, including its large and diverse sample size of young people from both in-school and out-of-school settings. This diversity offers a comprehensive overview of SRH literacy and service utilization across different social contexts, enhancing the representativeness of the findings within the study area. Additionally, the study employed a validated and well-established standardized multidimensional tool (Health Literacy Questionnaire) to comprehensively assess sexual health literacy, encompassing various domains of knowledge, skills, and attitudes, which enhances the reliability of the results. To the best of available evidence, this is the first study in the country to utilize such a robust and comprehensive tool for evaluating sexual health literacy among young people and comparing between in-school and out-of-school populations. This approach enhances the validity and applicability of the findings and sets a precedent for future research in this field.

### *Implication for Nursing Practice*

The low level of sexual and reproductive health literacy and service utilization demonstrated among young people across the study settings is a cause for concern. Nurses play a pivotal role in addressing these gaps, especially those working in schools, primary health centers, and youth-friendly clinics. Strengthening SRH literacy, especially for out-of-school youth and those with less formal education, requires nurses to deliver comprehensive, age-appropriate sexuality education and engage in culturally sensitive community outreach. Implementing comprehensive sexuality education, culturally sensitive outreach, and youth-friendly services could empower young people with the knowledge and tools they need to make informed health decisions. Such strategies are crucial for enhancing SRH outcomes and enabling young individuals to safely address their SRH needs. Also, nursing practice would benefit from further research using longitudinal approaches and expanded geographical coverage, including studies that test the impact of different interventions.

## Conclusion

The study reveals a significant gap in SRH literacy and service utilization among young people in the study area. Most participants demonstrated low SRH literacy levels, with only a small fraction accessing necessary SRH services, which was statistically significant.

## Supplemental Material

sj-docx-1-son-10.1177_23779608251411367 - Supplemental material for Sexual and Reproductive Health Literacy and Service Utilization Among Young People in Southwest Nigeria Supplemental material, sj-docx-1-son-10.1177_23779608251411367 for Sexual and Reproductive Health Literacy and Service Utilization Among Young People in Southwest Nigeria by Aanuoluwapo Omobolanle Olajubu, Abiola Olubusola Komolafe and Adesegun Olayiwola Fatusi in SAGE Open Nursing
