# Supplementary material for: Sexual and Reproductive Health Literacy and Service Utilization Among Young People in Southwest Nigeria
Source: SAGE Open Nurs. 2026 Jan 27;12:23779608251411367. doi: 10.1177/23779608251411367 (PMC12847649; doi:10.1177/23779608251411367)
Supplement: sj-docx-1-son-10.1177_23779608251411367 - Supplemental material for Sexual and Reproductive Health Literacy and Service Utilization Among Young People in Southwest Nigeria [file sj-docx-1-son-10.1177_23779608251411367.docx]

**STROBE Statement—Checklist:**

**SEXUAL AND REPRODUCTIVE HEALTH LITERACY AND SERVICE UTILIZATION AMONG YOUNG PEOPLE IN SOUTHWEST NIGERIA**

| **Section** | Item No | | Recommendation | Remark | Page Number |
| --- | --- | --- | --- | --- | --- |
| **Title and abstract** | 1 | | Indicate the study’s design with a commonly used term in the title ***or*** the abstract | **Done** | **1** |
|  |  |  | Provide in the abstract an informative and balanced summary of what was done and what was found | **Done** | **1** |
| Introduction | | | |  |  |
| Background/rationale | | 2 | Explain the scientific background and rationale for the investigation being reported | **Done** | **2-4** |
| Objectives | | 3 | State specific objectives, including any pre-specified hypotheses | **Done** | **4** |
| Methods | | | |  |  |
| Study design | | 4 | Present key elements of study design early in the paper | **Done** | **4** |
| Setting | | 5 | Describe the setting, locations, and relevant dates, including periods of recruitment, exposure, follow-up, and data collection | **Done** | **4** |
| Participants | | 6 | Give the eligibility criteria, and the sources and methods of selection of participants | **Done** | **4-6** |
| Variables | | 7 | Clearly define all outcomes, exposures, predictors, potential confounders, and effect modifiers. Give diagnostic criteria, if applicable | **Done** | **8** |
| Data sources/ measurement | | 8* | For each variable of interest, give sources of data and details of methods of assessment (measurement). Describe comparability of assessment methods if there is more than one group | **Done** | **8** |
| Bias | | 9 | Describe any efforts to address potential sources of bias | **Done** | **8** |
| Study size | | 10 | Explain how the study size was arrived at | **Done** | **6** |
| Quantitative variables | | 11 | Explain how quantitative variables were handled in the analyses. If applicable, describe which groupings were chosen and why | **Done** | **8-9** |
| Statistical methods | | 12 | Describe all statistical methods, including those used to control for confounding | **Done** | **9** |
|  |  |  | Describe any methods used to examine subgroups and interactions | **Not applicable** |  |
|  |  |  | Explain how missing data were addressed | **Not applicable** |  |
|  |  |  | If applicable, describe analytical methods taking account of sampling strategy | **Not applicable** |  |
|  |  |  | Describe any sensitivity analyses | **Not applicable** |  |
| Results | | | |  |  |
| Participants | | 13* | Report numbers of individuals at each stage of study—eg numbers potentially eligible, examined for eligibility, confirmed eligible, included in the study, completing follow-up, and analysed | **Done** | **9** |
|  |  |  | Give reasons for non-participation at each stage |  |  |
|  |  |  | Consider use of a flow diagram | **Not applicable** |  |
| Descriptive data | | 14* | Give characteristics of study participants (eg demographic, clinical, social) and information on exposures and potential confounders | **Done** | 9 |
|  |  |  | Indicate number of participants with missing data for each variable of interest | **Not applicable** |  |
| Outcome data | | 15* | Report numbers of outcome events or summary measures | **Done** |  |
| Main results | | 16 | Give unadjusted estimates and, if applicable, confounder-adjusted estimates and their precision (eg, 95% confidence interval). Make clear which confounders were adjusted for and why they were included | **Done** | **12-13** |
|  |  |  | Report category boundaries when continuous variables were categorized | **Not applicable** | **11** |
|  |  |  | If relevant, consider translating estimates of relative risk into absolute risk for a meaningful time period | **Not applicable** |  |
| Other analyses | | 17 | Report other analyses done—eg analyses of subgroups and interactions, and sensitivity analyses | **Not applicable** |  |
| Discussion | | | |  |  |
| Key results | | 18 | Summarize key results with reference to study objectives | **Done** | **17** |
| Limitations | | 19 | Discuss limitations of the study, taking into account sources of potential bias or imprecision. Discuss both direction and magnitude of any potential bias | **Done** | **23** |
| Interpretation | | 20 | Give a cautious overall interpretation of results considering objectives, limitations, multiplicity of analyses, results from similar studies, and other relevant evidence | **Done** | **17-23** |
| Generalizability | | 21 | Discuss the generalizability (external validity) of the study results | **Done** | **23** |
| Other information | | | |  |  |
| Funding | | 22 | Give the source of funding and the role of the funders for the present study and, if applicable, for the original study on which the present article is based | **Done** | **23** |
